# Relevance of Patient-Reported Outcome Measures in Patients with Cancer: Detection of Underrated Psychological Distress of Palliative Care Patients in an Outpatient Setting

**DOI:** 10.1089/pmr.2023.0075

**Published:** 2024-05-15

**Authors:** Madeleine Fink, Sandy Müller, Eva Warnecke, Jörg Hense, Martin Schuler, Martin Teufel, Maria Rosa Salvador Comino, Mitra Tewes

**Affiliations:** ^1^ Clinic for Psychosomatic Medicine and Psychotherapy, LVR-University Hospital Essen, University of Duisburg-Essen, Essen, Germany.; ^2^ Department of Psycho-Oncology, West German Cancer Center, National Center for Tumor Diseases (NCT), Essen, Germany.; ^3^ Center for Translational Neuro- and Behavioral Sciences (C-TNBS), University of Duisburg-Essen, Essen, Germany.; ^4^ Department of Palliative Medicine, West German Cancer Center, University Hospital Essen, University of Duisburg-Essen, Essen, Germany.; ^5^ Department of Medical Oncology, West German Cancer Center Essen, University Hospital Essen, Essen, Germany.; ^6^ National Center for Tumor Diseases (NCT) West, Essen, Germany.

**Keywords:** advanced cancer, Minimal Documentation System, outpatient, palliative care, patient-reported outcome measurement

## Abstract

**Background:**

The overall level of physical and psychological symptom burden of advanced cancer patients (ACP) in an outpatient setting is notoriously difficult to assess. Therefore, more efficient and objective assessment is needed to accomplish this important task.

**Objectives:**

The aim of this study was to compare the physical and psychological symptom burden rated by palliative care nurse (PCN) versus patient’s self-rating.

**Design:**

This retrospective German cohort study analyzed symptom burden using an electronic patient-reported outcome measure (ePROM). After referral to an outpatient specialized palliative care team, a PCN assessed the patient’s symptoms both up to three months before initial presentation (IP) and at IP.

Group differences were identified using analyses of variance (ANOVA). Further descriptive analysis of patient characteristics was used.

**Subjects:**

The study enrolled 164 ACP who were referred to a specialized palliative care (SPC) team. Mean age was 62 (± 12.6) years. Gastrointestinal (*n* = 46; 28.0%), lung (*n* = 32; 19.5%), and breast cancer (*n* = 34; 20.7%) were the most common entities.

**Results:**

Most frequent reasons for referral were pain (*n* = 55; 33.5%) and social care problems (*n* = 36; 22.0%). Patients reported significantly higher grades on depression (*n* = 144; *Z* = −2.8, *p* = 0.005), anxiety (*n* = 144; *Z* = −2.376, *p* = 0.018), and worsened general condition (*n* = 139; *Z* = −7.005, *p* < 0.001).

**Conclusion:**

ACP in an outpatient setting were more frequently referred to SPC for pain management and assistance with social problems with regard to the cancer and its limitations. Psychological distress was underrated by the PCN in comparison with patient self-reporting through ePROM. This underlines the importance of self-reported outcome measurement.

## Introduction

Early integration of palliative care (PC) into standard oncology practice is recommended by International Guidelines.^1–3^ To this end, detailed assessment and management of symptoms in advanced cancer patients (ACP) are important goals of PC. Therefore, assessment of symptoms and problems of ACP is recommended as part of the routine clinical practice.^4^ Currently, incorporation of patient-reported outcome measures (PROMs) in the routine health care is advised to recognize symptoms and their severity earlier, to highlight patient’s needs, and to monitor and intervene when problems arise.^5–7^ In addition, systematic monitoring using PROMs has been shown to improve patient well-being, symptom management, and communication between patients and clinicians, as well as overall survival.^8,9^ However, self-reported outcome measures are not yet being routinely integrated into clinical practice.^10^

Owing to continuous improvement of treatment options for ACP, cancer is increasingly becoming a “chronic disease” with longer survival times.^11^ In this context, outpatient care and treatment options such as specialized palliative care (SPC) are becoming more important.^12^ PROMs can be useful to visualize the patient’s individual needs and necessary treatments, which is an important goal in PC.

One of these PROMs, the Minimal Documentation System (MIDOS2),^13^ which is a German version of the Edmonton Symptom Assessment Scale (ESAS),^14^ is a reliable and valid assessment tool recommended by German S3-guidelines.^15^ Symptom burden is assessed quarterly in our outpatient tertiary oncology center using an electronic patient-reported outcome measure (ePROM), which includes MIDOS2. If symptom burden is high and/or if specifically requested, a referral to SPC by oncologists might be considered. At the time of the initial outpatient encounter, a palliative care nurse (PCN) evaluates the patient’s symptoms using MIDOS2.

By aligning views of nurses and physicians regarding outcome measurements in PC, PCNs frequently carry out these assessments in clinical settings.^16–18^ However recent research highlighted that crucial outcome parameters, such as pain and psychological distress, are largely underestimated by nursing staff.^19^ In this previous study, nurses did not rate pain and anxiety symptoms, whereas inpatients reported at least mild symptom distress. Furthermore, a previous study conducted at our institution identified depression, anxiety, and weakness as significant predictors for the request of an SPC referral among oncological inpatients.^20^

It is assumed that acquisition of data through ePROM can enhance quality of supportive and palliative care by improving transparency in access to psycho-oncological interventions, which can strengthen patient autonomy and adherence.^21^ In addition to the fact that PROM symptom scores (e.g., MIDOS) could lead straight to improved PC, by using psychometric inventories, they could also lead to a psycho-oncological contact according to a pathway model to assess whether more comprehensive psycho-oncological interventions are needed. Therefore, we hypothesize that patients’ psychological symptoms are particularly underrated when assessed by a PCN.

This study aims to assess whether there are variations in the evaluation of physical and psychological symptoms between external nurse-reported and self-reported assessment of ACP in an outpatient setting. It further emphasizes the relevance of PROMs in this setting and, more importantly, to optimize support for this vulnerable cohort.

## Methods

### Study design

For this retrospective, monocentric study, we conducted a comprehensive analysis of medical records of 164 outpatients referred to an outpatient SPC of a German Comprehensive Cancer Center between November 2013 and December 2020. The time of death was obtained from the local residents’ registration office. Documentation of patient’s characteristics at the time of their initial presentation (IP) to the SPC outpatient clinic was extracted from medical records. Moreover, the MIDOS2 questionnaire was provided to be filled out by patients at IP. In addition, the MIDOS2 questionnaire was completed within a maximum of three months before IP. Notably, three experienced PCNs were involved in patient care during the observation period and were responsible to complete nurse-reported symptom assessment.

### Demographics

Participants had to meet the following inclusion criteria at IP to SPC: age older than 18 years and a histologically confirmed cancer type, stage IV according to *the Union for International Cancer Control* (*UICC*). In addition to demographic information such as sex, age, cancer diagnosis, date of first diagnosis, date of metastasis, and the reason for referral to SPC listed by the attending physician, we collected information from the PROM “MIDOS2,” which is integrated in the HOPE Symptoms and Problem Checklist and completed by the PCN at IP.

### Questionnaires

MIDOS2 is a validated tool for self-assessment symptom burden in APC. The questionnaire consists of 10 items about current symptoms, including pain, nausea, vomiting, dyspnea, constipation, weakness, loss of appetite, tiredness, depression, and anxiety. In addition, patients rate their subjective general condition. Symptoms are scored on a 4-point Likert scale (0 = no, 1 = mild, 2 = moderate, and 3 = severe).^13,22^

The German Hospice and Palliative Care Evaluation Symptoms and Problem Checklist (HOPE) is a validated and commonly used tool used for documentation in German inpatient and outpatient hospice and PC services.^23,24^ This checklist includes 16 different items: 8 for physical symptoms (pain, nausea, vomiting, dyspnea, constipation, weakness, loss of appetite, and tiredness), 2 special nursing problems (wound care and assistance with activities of daily living [ADLs]), and 4 psychological issues (depression, anxiety, confusion, and tension), and 2 social topics (organization of care and burden on family). Symptoms were assessed using a 4-point-Likert scale (0 = none, 1 = mild, 2 = moderate, and 3 = strong).^25^

### Statistical analyses

Data management and analyses were conducted using Statistical Program for Social Sciences version 29.0 (IBM, NY). To characterize the patients at first, SPC referral descriptive statistics were used. A one-sample *t* test was utilized to compare this study cohort with the cohort from Stiel et al.^13^ Group differences between patient-reported outcomes and observer-reported levels were assessed using the Mann–Whitney U test in case of non-normal distributions. Furthermore, we used repeated-measures analysis of variance (ANOVA) to analyze differences in symptom burden over time. The significance level was set at *p* < 0.05 for all statistical tests. Standard deviation is given in parentheses later.

## Results

### Patient characteristics

Between November 2013 and December 2020, a total of 281 patients were referred to the SPC. Among them, 164 had completed the MIDOS2 questionnaire within three months before IP in the oncological outpatient clinic. The mean age was 62 years (± 12.49) at IP.

Most participants were female (*n* = 97, 59.1%). The most frequent tumor entities were gastrointestinal (*n* = 46, 28%), lung (*n* = 32, 19.5%), and breast cancer (*n* = 34, 20.7%). Approximately half of the patients were initially diagnosed with metastatic cancer, categorized as UICC IV (*n* = 84, 52.4%). The time between the first diagnosis and IP was on average 4 months (± 5.44) and 7.4 months before death (± 9.31). Pain management (*n* = 55, 33.5%) and social care planning (*n* = 36, 22%) were the main reasons for IP to the SPC. At the time of IP, 57% of the patients still received chemotherapy: 42 of 164 patients (25.6%) received a first-line chemotherapy and 47 patients (28.7%) received a second-line chemotherapy. A total of 53% of the ACP had previously requested a referral to SPC. Table 1 shows the main characteristics of all outpatients at IP.

**Table 1.** Characteristics of Outpatients Referred for the First Time to Specialized Palliative Care

Patient characteristics	Total, *N* (%)
Number of patients	
Gender	**164**
Male	67 (40.9)
Female	97 (59.1)
Age at first presentation, in years	
Mean ± SD	61.6 (12.49)
Site of primary tumor	**164**
Gastrointestinal tract	46 (28.0)
Lung	32 (19.5)
Breast	34 (20.7)
Sarcoma	23 (14.0)
Genitourinary	4 (2.4)
Head and neck	9 (5.5)
Others^b^	16 (9.9)
Months after first metastasis to first SPC referral	
Mean ± SD	29.6 (33.71)
Months from first SPC referral to death	
Mean ± SD	7.4 (9.31)
Months from first diagnosis to first SPC referral	
Mean ± SD	4.01 (5.44)
Chemotherapy at the time of first SPC referral	**164**
Yes	124 (75.6)
No	40 (24.4)
Chemotherapy line	**164**
1st	42 (25.6)
2nd	47 (28.7)
3rd	30 (18.3)
>3rd	30 (18.3)
None	15 (9.1)
Previous patient’s request for SPC	**134^a^**
Yes	71 (53.0)
Yes, but later	23 (17.1)
No	40 (29.9)
Reason for SPC referral	**164**
Pain	55 (33.5)
Social care planning	36 (22.0)
Dyspnea	11 (6.7)
Nutritional advice	9 (5.5)
Psychological distress	14 (8.5)
Fatigue	4 (2.4)
More than one reason^c^	35 (21.4)

^a^ Subgroup: Patients who have filled out the minimal documentation system (MIDOS2) before their first SPC encounter.

^b^ Others: Include hematologic, central nervous system, malignant melanoma, cancer unknown primary (CUP), other gynecologic than breast cancer.

^c^ Includes other reasons such as physiotherapy, edema treatment, itching, incontinence, wound management, and constipation.

SD, standard deviation; SPC, specialized palliative care.

### Group difference regarding MIDOS2

**FIG. 1.**
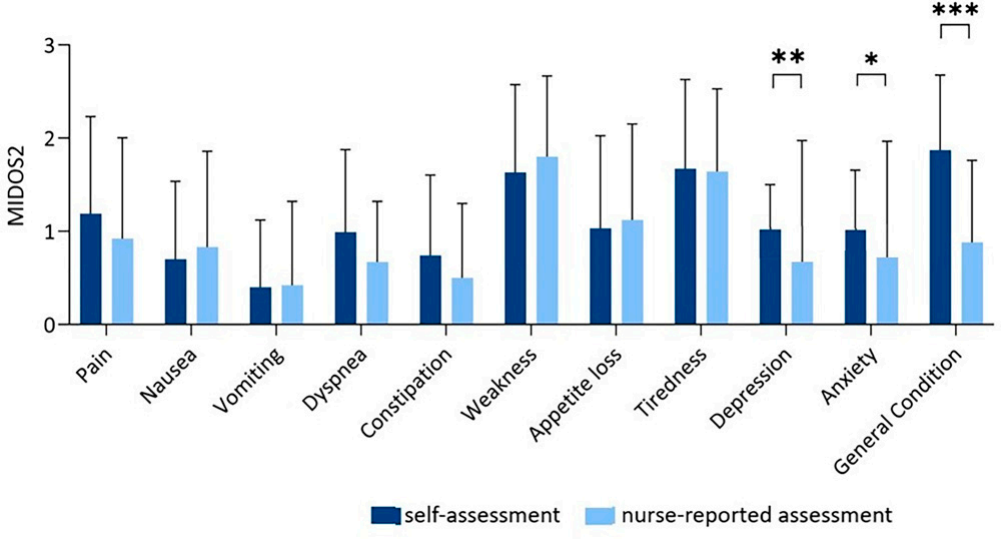
Patient’s self-assessment regarding symptom burden using the Minimal Documentation System in comparison with nurse-reported assessment. Note: *p < 0.05; **p < 0.01; ***p < 0.001.

At the time of IP, 70.5% of the patients were in a moderate or worsened general condition (*n* = 139) measures with ECOG. The most frequently reported moderate and severe symptoms were tiredness (*n* = 90/148, 60.8%), weakness (*n* = 81/147, 55.1%), loss of appetite (*n* = 51/147, 34.7%), and pain (*n* = 62/145, 42.76%). The symptom assessment conducted by the PCN at IP was comparable to those reported by the patients (Fig. 1).

In contrast, the comparison between self-reported symptoms and nurse-reported symptoms demonstrated a significant difference in grading of depression (*n* = 144; *Z* = −2.8, *p* = 0.005), anxiety (*n* = 144; *Z* = −2.376, *p* = 0.018), and subjective general condition (*n* = 139; *Z* = −7.005, *p* < 0.001). Patients rated the severity of these symptoms higher than the PCN did. There was no difference regarding the other symptoms (see Supplementary Table S1).

## Discussion

In this retrospective study, we analyzed data of ACP referred to SPC from a large comprehensive cancer center to assess symptom burden. In parallel, we explored differences between nurse-reported and self-reported assessment through ePROM.

Our findings indicate that the studied group of ACP referred to SPC suffers from a wide range of complex physical symptoms and moderate to high psychological distresses. Specifically, weakness, tiredness, loss of appetite, pain, and subjective poor general well-being were common, similar to previous studies analyzing patients with cancer at IP in an SPC.^26^ Moreover, patients themselves rated psychological symptoms such as anxiety and depression significantly higher than PCNs. These findings are in line with previous studies, which have demonstrated a tendency for clinicians to underestimate symptom intensity in patients with cancer.^27,28^

Xiao et al. described that providers especially underestimated the incidence, severity, and distress of symptoms experienced by ACP. The discrepancies became even more apparent when symptoms were more severe and distressing. A cross-sectional study by Laugs et al. included 1933 patient-health care provider dyads. The study found that patients, especially those with low Karnofsky Performance Status and high Mini Mental State Score, and who were hospitalized, recently diagnosed, or undergoing opioid titration, were at increased risk of having their symptoms underestimated by health care providers.

Literature showed that various general and specific reasons have been proposed to explain the discrepancies on symptom assessment between clinicians and patients.^26^ General factors include, for example, communication and gender. Studies have shown that improved communication between patients and physicians is associated with higher patient-reported symptom counts.^29,30^

In addition, physicians tend to perceive more symptoms in their patients when there is better communication. For example, oncologists were two to five times more likely to identify depression in their patients if they had a deeper understanding about their patients.^31^ The findings of our investigation underline this point. If there is not enough time for in-depth assessment during outpatient referral, which is not uncommon in an outpatient setting, communication appears to suffer as a consequence. Affective symptoms are therefore apparently less well detected. In a similar sense, gender also appears to play a role.^31^ Although this issue was not observed in our cohort, it has been included as a subject for future research.

Overall, it can be assumed that not all contributing factors may have been identified yet.^26^ Thus, patients’ self-assessment can provide a distinct benefit.^32^ This is one of the reasons why PROMs are currently widely recommended. Our study demonstrates their superiority in detecting affective symptoms for the presented cohort.

Moreover, there is growing evidence suggesting that PROMs offer independent prognostic information for overall survival.^33^ In a systematic review and meta-analysis by Efficace et al., strong evidence was found that PROMs provide independent prognostic information for overall survival across different cancer populations and disease stages. Although the precise mechanisms underlying the association between PROMs and survival remain unclear, this study’s results provide evidence that self-reported health status data offer unique prognostic insights not captured by conventional clinical assessments. Similar conclusions have been drawn in studies involving the general population.^34^ So far, there has been a lack of understanding of the mechanisms involved. It remains to be speculated whether the better detection of affective symptoms and the resulting supposedly better psycho-oncological and palliative support, in accordance with these data, might provide an additional benefit.

However, sometimes patients are unable to self-report their symptoms because of factors such as high symptom load, language barriers, and limited ability to use electronic devices, as well as physical, cognitive, or neurological disabilities.^35,36^ In case of such limitations, it is important to recognize that relying solely on PROMs as an only source of information may not be appropriate for every patient subgroup, as this may result in a lack of comprehensive clinical data. Therefore, we recommend the use of health care providers to support these patient collectives individually, a practice advocated in previous studies.^19,37^

Psychological problems and overall well-being exhibited a significant difference between self-assessment and nurse-reported assessment, with patients being underrated by PCN in our cohort of outpatients. Comparable results could be observed in inpatients.^19^ These differences highlight the central role of PROMs in the daily routine of ACP, as well as PC clinics. Importantly, integrating PROMs into clinical practice does not require additional time expenditure, as demonstrated by the same study. Although there is a limited body of research in this particular field of psycho-oncology,^7^ it is becoming increasingly evident that empowering health care professionals to utilize PROM data in patient diagnosis and treatment can lead to improved psycho-oncological support and relief of psychological distress in particular, as PROMs capture highly clinically relevant information.

Our results underline the benefits of PROMs, particularly in the case of psychological distress. However, the results also emphasize the equivalence of the use of nursing assessments in everyday clinical practice when it concerns the physical assessment of care needs. In addition to providing better, patient-oriented support for psychological distress in this vulnerable group in everyday clinical practice, PROMs could lead to higher autonomy and patient control.^21^

## Limitations

Our study has several limitations. The retrospective nature of the study prevents us from drawing firm conclusions. Data are from a single Comprehensive Cancer Center and may not be generalizable. Data were limited to those available from our database, and MIDOS2 scores were not available or complete for all patients. In addition, the period between the self-completed questionnaires from the outpatient clinic at IP varied largely (up to three months), potentially skewing our data. Further prospective studies are warranted in this regard.

## Conclusion

The first referral of ACP to an SPC consultation is usually for pain management or social issues. In particular, the symptoms of depression, anxiety, and general well-being are significantly underestimated in the external nurse-reported assessment compared with the patient’s self-assessment. For many patients, the combination of external and self-reported assessments might be complementary and needed. Further and larger studies in this area are needed.

## Ethics Approval and Consent to Participate

All procedures performed in studies involving human participants were in accordance with the ethical standards of the institutional and local ethics review committee of the University of Duisburg-Essen and approved the data analysis (16–6800-BO) and were in accordance with the 1964 Declaration of Helsinki and its later amendments or comparable ethical standards.

## Supplementary Material

Supplementary Table S1

## Data Availability

The datasets used and analyzed during this study are available from the corresponding author on reasonable request.

## Abbreviations Used

ANOVA: analyses of variance
ACP: advanced cancer patients
ePROM: electronic patient-reported outcome measure
ESAS: Edmonton Symptom Assessment Scale
HOPE: The German Hospice and Palliative Care Evaluation Symptoms and Problem Checklist
IP: initial presentation
MIDOS2: Minimal Documentation System
PC: palliative care
PCN: palliative care nurse
PROM: Patient reported Outcome Measurement
SPSS: Statistical Program for Social Sciences
UICC: Union for International Cancer Control
