# Supplementary material for: Relevance of Patient-Reported Outcome Measures in Patients with Cancer: Detection of Underrated Psychological Distress of Palliative Care Patients in an Outpatient Setting
Source: Palliat Med Rep. 2024 May 15;5(1):194–200. doi: 10.1089/pmr.2023.0075 (PMC11265614; doi:10.1089/pmr.2023.0075)
Supplement: Supplementary Table S1 [file pmr.2023.0075_supplement_table2.docx]

**Supplements**

**Table 2. Patient’s self-assessment regarding symptom burden using MIDOS2 in comparison (ANOVA) to nurse-reported assessment**

| **Predictor** | **n** | **Mean_Patient_ (SD)** | **Mean_Nurse_ (SD)** | **Z** | **p-value** |
| --- | --- | --- | --- | --- | --- |
| pain | 145 | 1.19 (1.041) | 1.26 (0.955) | -1.0 | 0.317 |
| nausea | 155 | 0.68 (0.865) | 0.71 (0.822) | -0.378 | 0.705 |
| vomiting | 133 | 0.27 (0.645) | 0.41 (0.693) | -0.707 | 0.480 |
| dyspnoea | 153 | 0.91 (0.915) | 0.99 (0.807) | -1.134 | 0.257 |
| constipation | 147 | 0.72 (0.897) | 0.66 (0.772) | -0.905 | 0.366 |
| tiredness | 147 | 1.63 (0.944) | 1.75 (0.848) | -0.824 | 0.410 |
| Loss of appetite | 147 | 1.03 (0.996) | 1.08 (1.009) | -0.098 | 0.922 |
| weakness | 148 | 1.67 (0.958) | 1.57 (0.891) | -1.087 | 0.277 |
| depression | 144 | 0.92 (0.927) | 0.78 (0.815) | -2.8 | 0.005 |
| anxiety | 144 | 1.02 (0.942) | 0.82 (0.872) | -2.302 | 0.021 |
| general condition* | 139 | 1.87 (0.806) | 0.9 (0.882) | -7.005 | <0.001 |

*inverted
